# The Female Lower Genital Tract Is a Privileged Compartment with IL-10 Producing Dendritic Cells and Poor Th1 Immunity following *Chlamydia trachomatis* Infection

**DOI:** 10.1371/journal.ppat.1001179

**Published:** 2010-11-04

**Authors:** Ellen Marks, Miguel A. Tam, Nils Y. Lycke

**Affiliations:** Department of Microbiology and Immunology, Mucosal Immunobiology and Vaccine Center, Institute of Biomedicine, The University of Gothenburg, Gothenburg, Sweden; Yale University School of Medicine, United States of America

## Abstract

While a primary genital tract infection with *C. trachomatis* stimulates partial-protection against re-infection, it may also result in severe inflammation and tissue destruction. Here we have dissected whether functional compartments exist in the genital tract that restrict Th1-mediated protective immunity. Apart from the Th1-subset, little is known about the role of other CD4^+^ T cell subsets in response to a genital tract chlamydial infection. Therefore, we investigated CD4^+^ T cell subset differentiation in the genital tract using RT-PCR for expression of critical transcription factors and cytokines in the upper (UGT) and lower genital tract (LGT) of female C57BL/6 mice in response to *C. trachomatis* serovar D infection. We found that the Th1 subset dominated the UGT, as IFN-γ and T-bet mRNA expression were high, while GATA-3 was low following genital infection with *C. trachomatis* serovar D. By contrast, IL-10 and GATA-3 mRNA dominated the LGT, suggesting the presence of Th2 cells. These functional compartments also attracted regulatory T cells (Tregs) differently as increased FoxP3 mRNA expression was seen primarily in the UGT. Although IL-17A mRNA was somewhat up-regulated in the LGT, no significant change in RORγ-t mRNA expression was observed, suggesting no involvement of Th17 cells. The dichotomy between the LGT and UGT was maintained during infection by IL-10 because in IL-10-deficient mice the distinction between the two compartments was completely lost and a dramatic shift to the predominance of Th1 cells in the LGT occurred. Unexpectedly, the major source of IL-10 was CD11c^+^ CD11b^+^ DC, probably creating an anti-inflammatory privileged site in the LGT.

## Author Summary

The immune response to the genital tract pathogen *C. trachomatis* can result in a number of pathological outcomes including tubal scarring and consequently, infertility. CD4^+^ T helper 1 (Th1) cells are critical for host protection against infection, but may also contribute to immunopathology. Apart from the Th1 cells, little is known about the role of other CD4^+^ T cell subsets in response to a genital tract chlamydial infection. By tracking the development of T helper cells in the genital tract using RT-PCR for distinct transcription factors associated with these subsets, we found vastly different immune responses in the upper genital tract (UGT) compared to the lower genital tract (LGT) of female mice during infection. The LGT was dominated by anti-inflammatory IL-10 production from dendritic cells (DC) and the non-protective Th2 subset. In contrast, the upper genital tract was populated by protective-Th1 cells. In the absence of IL-10, though, the LGT and UGT were both dominated by Th1 cells, arguing that DC-derived IL-10 secures an anti-inflammatory privileged site in the LGT. These findings provide a break-through in our understanding of functional compartments in the genital tract immune system with potentially strong impact on vaccine development.

## Introduction

*Chlamydia trachomatis* is an intracellular bacterium that infects the genital and ocular mucosae. The genital tract infection is the number one cause of bacterial sexually transmitted disease (STD) world-wide. Because the infection is asymptomatic in up to 70% of females and can result in severe damage of the reproductive tract, it is one of the major causes of tubal factor infertility [1]. It is generally agreed that the best protection against infection and sequelae could be achieved by an effective vaccine. However, vaccine development has been hampered by our poor understanding of protective immune mechanisms in the genital tract. In particular, the dichotomy between effector and regulatory functions that, on the one hand eliminate infection and on the other, could prevent immunopathology from developing, is inadequately defined for genital tract chlamydial infections.

It is known that chlamydial infection of the genital tract stimulates a complex array of host innate and adaptive immune responses. Cells of the innate immune system react rapidly to recognize and limit the infection, and ultimately influence the outcome of infection through the modulation of the adaptive immune response. Studies have shown that CD4^+^ T cells and Th1-cells, in particular, are necessary for the effective clearance of *Chlamydia* from the genital tract [2], [3], [4], [5], [6]. Protective immune responses to other infections such as herpes simplex virus-2 (HSV-2) and *Leishmania,* are also Th1-mediated and critically dependent on IFN-γ [7], [8]. However, we know that activated CD4^+^ T cells differentiate into a number of T helper subsets, including: T-helper 1 (Th1), Th2, Th17 and several subsets of T regulatory (Treg) cells, each subset capable of secreting a distinct cytokine profile (reviewed [9]). Differentiation of Ag-primed CD4^+^ T cell subsets is critically dependent on the cytokine milieu that regulates CD4^+^ T cell subset differentiation (reviewed [10]). Early on after cognate interaction with an antigen-presenting cell (APC), a developmental program mediated by a group of enzymes known as transcription factors is activated in the T cells. This enzymatic activity results in the removal of covalent modifications from histone tails, and together with DNA methylating enzymes, activates selected cytokine genes. This process allows for the expression of a signature profile of cytokine genes specific for that T cell subset. At the same time it silences the expression of cytokines and transcription factors of the opposing subsets, thereby resulting in lineage restriction [11]. Although not exclusively expressed in CD4^+^ T cells, expression of the transcription factors T-bet, GATA-3, RORγ-t and FoxP-3 can be used to identify Th1, Th2, Th17 and some Treg subsets, respectively [12], [13], [14], [15]. Th1 cells are associated with the production of IFN-γ and strong cell-mediated immunity, which is thought to be the primary mechanism for clearance of *Chlamydia* from the genital tract and protection against reinfection [3], [5]. The Th2 subset is associated with secretion of the cytokines IL-4, IL-5, IL-13 and antibody production, and is not effective in the defense against *Chlamydia* [16], [17]. Studies suggest that Th2-driven antibody production is of subordinate importance during a primary chlamydial infection, although it may contribute to protection against re-infection [18]. The Th17 subset has been ascribed critical roles in several infection models and autoimmune diseases, through the production of IL-17 and IL-23. Recently, it was demonstrated that lung chlamydial infections were dependent on IL-17 for Th1 protection to develop [19]. However, presently we do not know whether IL-17 or Th17 cells play any protective role in genital tract chlamydial infections [20], [21].

Advances in our understanding of the immunobiology of the genital tract and better knowledge about CD4^+^ T cell subset immunity in response to genital tract chlamydial infections are critical elements for the development of effective vaccines. Treg cell subsets have been ascribed a dampening function on inflammation. Tregs can be divided into the naturally occurring Tregs and inducible Tregs (iTreg), according to cell surface markers and cytokine producing abilities, primarily IL-10 or TGF-β. Tregs have been documented in the context of many bacterial infections, including intracellular infections with *Salmonella typhimurium* [22]. However, the analysis of Treg subsets and their actions in the genital tract mucosa are few, and the role of Tregs in chlamydial infections is still quite unclear. We recently reported that ICOS-deficient mice, have impaired Treg-development in response to *C. trachomatis* genital tract infection, which resulted in significantly augmented local inflammation and a more effective clearance of bacteria compared to that found in wild-type (WT) mice [23]. The relative roles of Th1, Th2, and to a lesser extent, Th17 cells, have been described for other bacterial infections [21]. However, their contribution to resistance and immunopathology against a genital tract chlamydial infection is incompletely known. Therefore, we undertook the present study to gain insight into the development of different effector and regulatory CD4^+^ T cell-subsets in response to a genital tract infection in mice with *C. trachomatis* serovar D. More specifically, we sought to understand the balance between effector and regulatory CD4^+^ T cell subsets in genital tract protective immunity and whether any distinct functional anatomical compartments could be identified.

## Results

### Infection induces striking CD4^+^ T cell infiltration in the genital tract and results in protective immunity against reinfection

**Figure 1:**
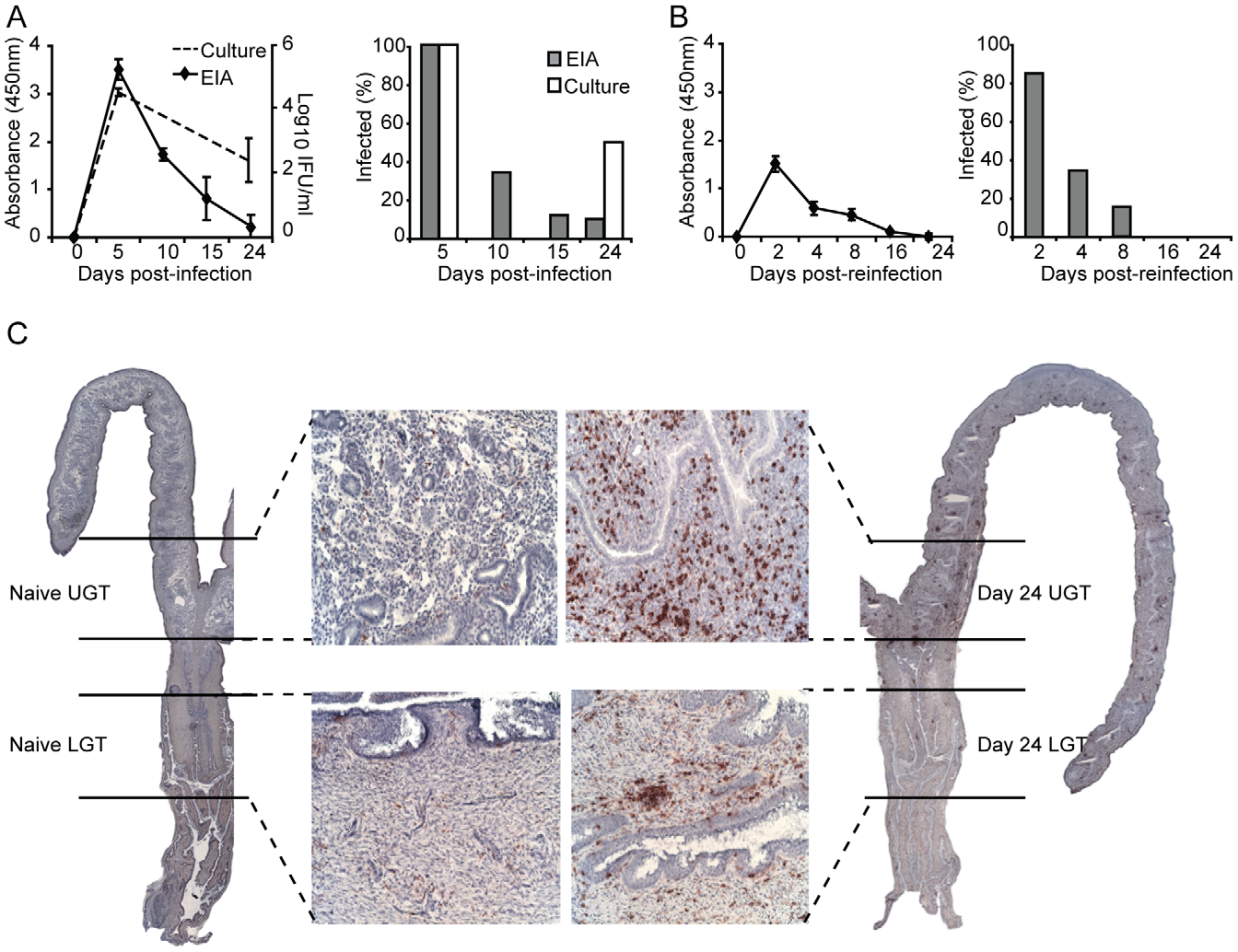
The adaptive immune response is protective against re-infection with *C. trachomatis*. (A) Bacterial shedding was determined at given intervals after a primary infection or (B) post-reinfection with *C. trachomatis*. Results are expressed as mean absorbance ± SEM as determined by *Chlamydia* Mikrotrak EIA, or log_10_ IFU as determined by culture or cervicovaginal swabs (left panels). Right panels, results are expressed as the percentage of animals infected within the group as determined by EIA or culture. (C) Whole sections of the genital tract indicating the dissection definition of upper (uterus and uterine horns; UGT) and lower (cervix and proximal vaginal tissue; LGT) genital tract. Representative sections are shown from naïve mice (left panel), and after 24 days of infection (right panels), stained for CD4^+^ T cells with 20× inset. Data is from one representative experiment of 2 or 3 giving similar results, each experiment including at least 15 mice per group.

It has previously been shown that a primary infection with *C. trachomatis* results in partially protective immunity against reinfection with the same serovar [24]. Following intravaginal infection, we found that peak shedding occurred between 5 and 10 days after infection. Importantly, by day 10, clearance of the infection in the genital tract had begun, and was completely eliminated by day 32 (Fig. 1A). To secure that the EIA-method used for detection of EBs reflected an ongoing infection we assessed inclusion forming units (IFU) in samples taken at some critical time points (Fig. 1A). This analysis demonstrated good correlation with the EIA detection method, albeit assessment of IFU was more sensitive at later time points, showing a higher level of infected mice compared to the EIA-method (Fig. 1A). In agreement with previous work, highly immune mice exhibited strong resistance against reinfection, suffering only a transient infection, with less than 40% of animals infected after 4 days (Fig. 1B) [24]. We and others have shown that CD4^+^ T cells are crucial for clearance of a primary infection with *C. trachomatis* from the genital tract and the development of protective immunity [3], [6], [25]. In accordance, intense CD4^+^ T cell infiltration during infection can be seen throughout the genital tract (Fig. 1C). In order to investigate CD4^+^ T cell differentiation during infection, we carefully dissected the UGT, which consisted of the uterus and uterine horns, from the cervix and proximal vaginal tissue of the LGT, for analysis by RT-PCR (Fig. 1C). Throughout the study this anatomical distinction was kept, separating UGT from LGT.

### Distinct and different cytokine dominance in the upper and lower female genital tract

**Figure 2:**
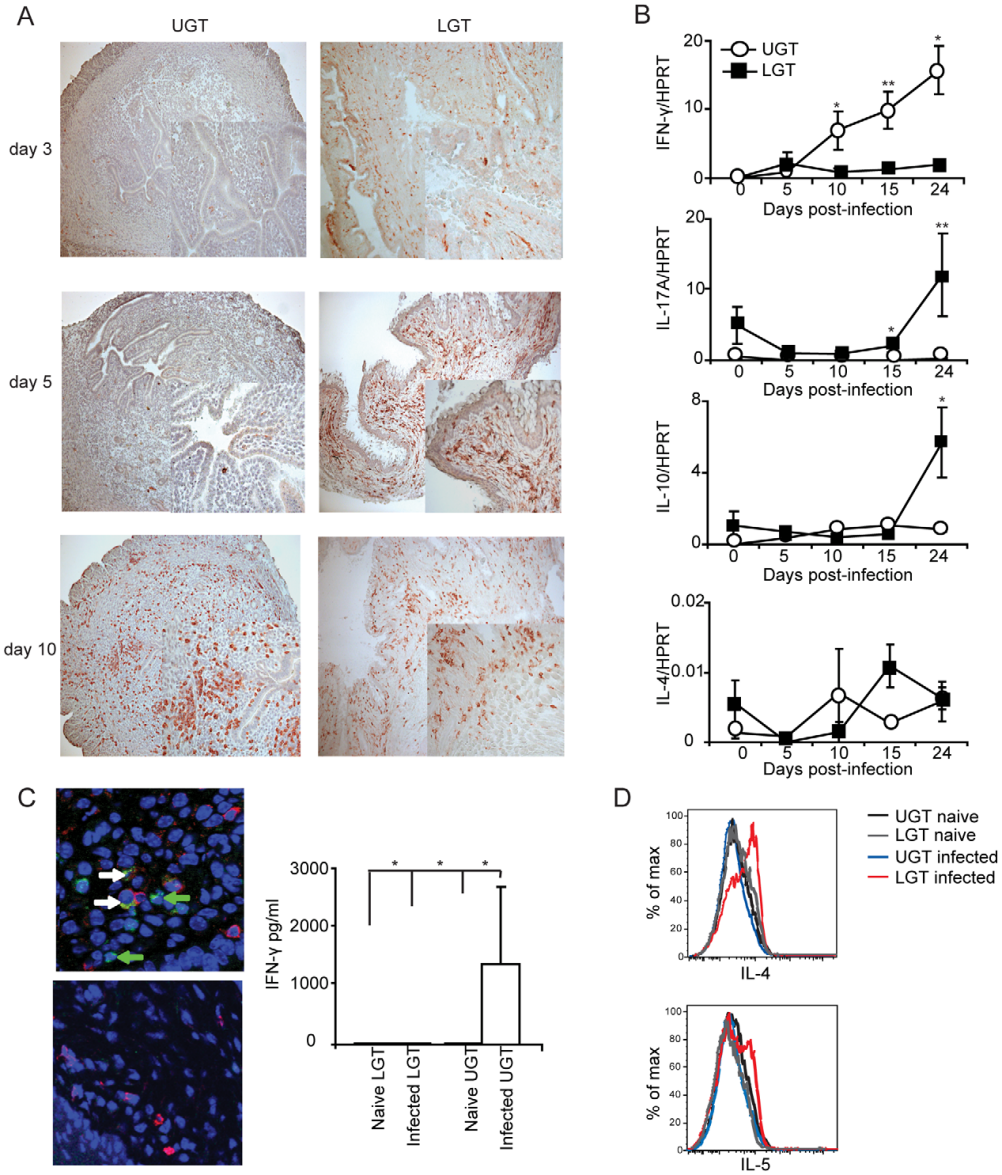
Distinct cytokine mRNA profiles in different regions of the genital tract during *C. trachomatis* infection. (A) sections of upper genital tract (UGT; left panels) and lower genital tract tissue (LGT; right panels) were stained for CD4^+^ T cells on day 3, day 5, and day 10 post-infection, as indicated. Representative sections from one experiment of 3 giving similar results are shown. Large photographs are 10× magnification with 20× inset. (B) Cytokine mRNA levels were determined using RT-PCR, in the LGT and UGT at the indicated time points of infection. Results are expression of IFN-γ, IL-17A, IL-10 and IL-4 mRNA normalized to the housekeeping gene, HPRT, ± SEM. Data is from a representative experiment out of 3 giving similar results, and 5 mice per time point. (C) Tissue sections from the UGT (upper panel) and LGT (lower panel) were stained for CD4 (TxRd; red), IFN-γ FITC; green) and Topro-3 (blue) on day 24 of infection. Cytokine bead array analysis was used to quantify the production of IFN- γ following PMA/ionomycin *in vitro* stimulation of CD4^+^ sorted T cells from infected mice on day 24 (right panel). (D) PMA/ionomycin stimulated CD4^+^ T cells from the LGT were stained for IL-4 (upper panel) or IL-5 (lower panel) and analysed by flow cytometry.* p<0.05, ** p<0.01.

**Figure 3:**
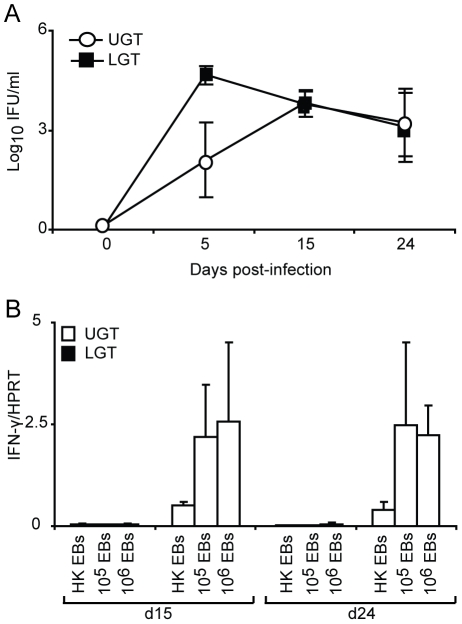
The dichotomy between upper and lower genital tract remains regardless of infectious dose. (A) The chlamydial inclusion forming unit (IFU) load in the upper (UGT) versus lower (LGT) genital tract was determined by culture of tissue homogenates on day 5, 15 and 24 of infection. Results are expressed as mean log_10_IFU/ml ± SEM from 4 mice per time point. (B) Mice were infected with 10^6^, 10^5^ *C. trachomatis* EBs or mock-infected with 10^6^ heat-killed (HK EBs). IFN-γ mRNA expression levels in the LGT and UGT on day 15 and 24 of infection were determined using RT-PCR. Results are expressed as mean expression normalized to the housekeeping gene, HPRT, ± SEM from 4 mice per time point.

Little is known about the kinetics of differentiation of T cell subsets in the genital tract during infection. CD4^+^ T cells in the early phase of the infection were more frequent in the LGT than in the UGT. However, by day 10 of the infection CD4^+^ numbers had begun to increase dramatically in the UGT (Fig. 2A). Parallel to this we found increases in mRNA expression of several important cytokines. Whereas increases in IFN-γ mRNA expression were seen from day 10 of infection in the UGT, little change was observed in the LGT (Fig. 2B). This pattern was also confirmed at the protein level by labeling of IFN- γ in frozen sections or production of IFN-γ by isolated CD4^+^ T cells after stimulation with PMA/ionomycin in UGT, but not by CD4+ T cells of the LGT, of infected mice (Fig. 2C). By contrast, IL-10 mRNA was increased in the LGT, in particular, with high expression levels recorded on day 24 (Fig. 2B). Hence, the LGT and UGT were characterized by distinct and different cytokine responses, with IFN-γ expression in the UGT and IL-10 dominating the LGT (Fig. 2B). Comparatively weak expression of mRNA for IL-4 was recorded in both UGT and LGT, but the latter showed higher levels on day 15 following infection (Fig. 2B, 2D). Evidence of Th17 subset activity through IL-17A mRNA expression was found predominantly in the LGT at the later time points of infection (Fig. 2B). The contrasting cytokine profiles of the UGT and LGT suggested that these compartments could be functionally different and subjected to unique regulatory control allowing different developmental or selection processes for the CD4^+^ T cell subsets. To rule out that the functional dichotomy observed between the UGT and LGT was due to differences in antigen/infectious load we followed the IFU in the respective tissue. Detection of IFUs in the UGT and LGT over time reflected an ascending infection and comparable antigen loads (IFUs) in the UGT and LGT (Fig. 3A). To confirm this, we challenged the mice with different doses of EBs or heat-killed EBs and we observed a similar dominance of Th1 cells in the UGT and practically no IFN-γ in the LGT irrespective of the challenge dose (Fig. 3B).

### Strictly regulated Th1 and Th2 activity in the upper and lower genital tract in response to a primary chlamydial infection

**Figure 4:**
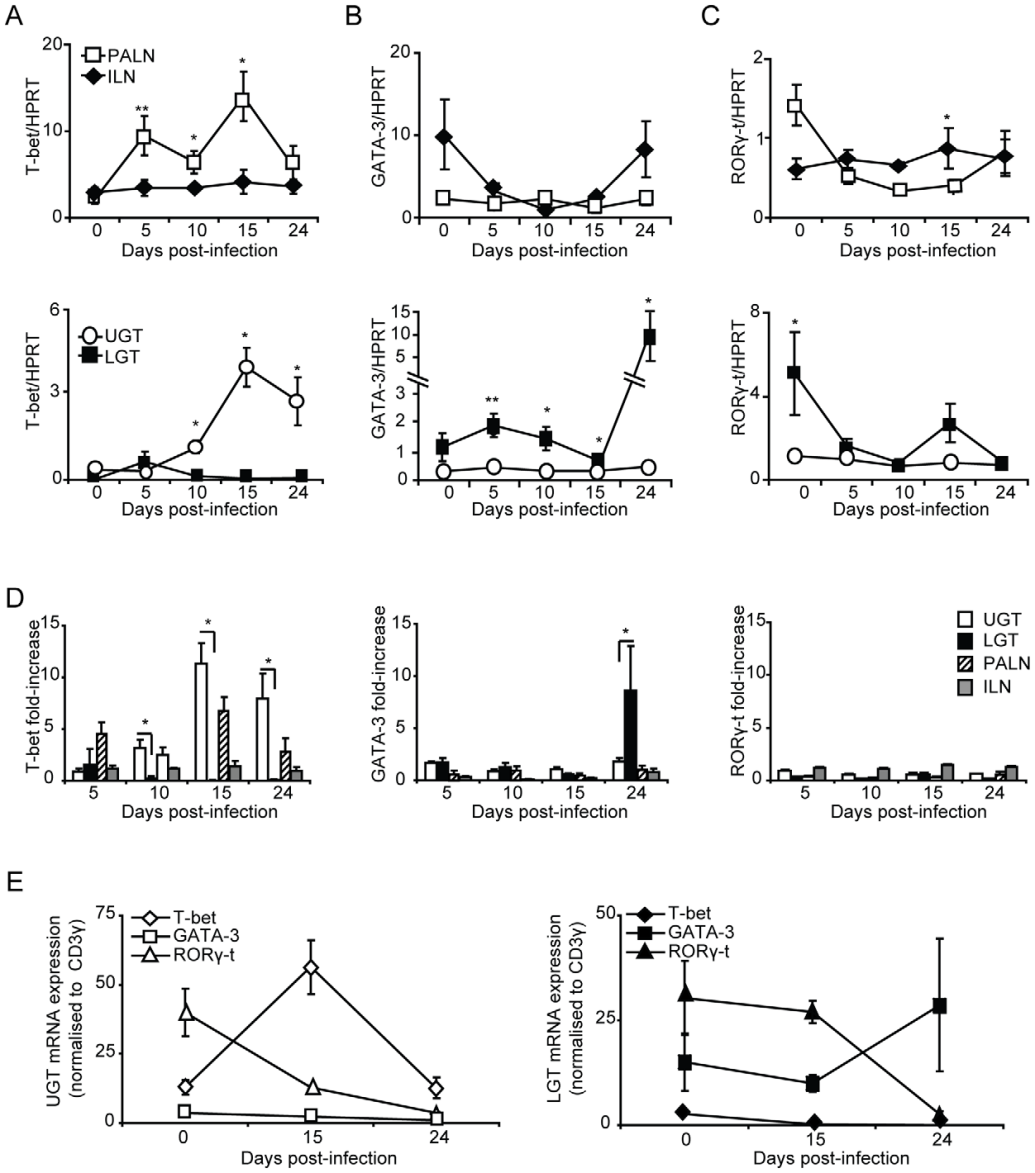
T-bet dominates the upper genital tract, while GATA-3 is upregulated in the lower genital tract. Regional lymph nodes (PALN and ILN), lower (LGT) and upper (UGT) genital tracts were collected from naïve and *C. trachomatis* infected mice at the indicated time points. RT-PCR was undertaken to determine the mRNA levels of (A) T-bet, (B) GATA-3 and (C) RORγ-t expression, normalized to the housekeeping gene (A-D) HPRT or CD3-γ (E). (A-C,E) Data is expressed as normalized mean expression ± SEM, or (D) fold-increase over naïve levels. Values are from one representative experiment of 3 giving similar results, and 5 mice per time point. * p<0.05, ** p<0.01.

To further investigate the nature and location of CD4^+^ T cell subsets during infection, we undertook RT-PCR analysis of T helper cell differentiation by monitoring transcription factor mRNA expression in the genital tract. The transcription factor T-bet has been shown to be the master regulator of CD4^+^ T differentiation into Th1-type cells, which controls the expression of IFN-γ, in addition to silencing T cell transcription factors of opposing T helper-subsets [14]. The activity of T-bet in the PALN compared to the ILN was higher at all measured time points during infection (Fig. 4A). Expression of T-bet mRNA in the UGT increased to a peak on day 15 (Fig. 4A), representing an 11-fold increase of expression from levels in naïve tissue (Fig. 4D). In striking contrast, T-bet mRNA expression was not increased in the LGT following infection (Fig. 4A). Furthermore, the transcription factor GATA-3, essential for Th2 differentiation, peaked later than T-bet transcription on day 24 with a 9-fold increase in mRNA for GATA-3 exclusively up-regulated in LGT and not in the UGT (p<0.05) (Fig. 4B,D). Of note, in naïve and infected mice GATA-3 mRNA expression was higher in the ILN than in the PALN, which contrasted with the pattern seen for T-bet mRNA expression (Fig. 4B, D). These expression patterns for Th1 and Th2 activity was also obtained when the transcription factor mRNA expression was normalized against CD3-□ mRNA levels in the respective tissues (Fig. 4E). Thus, we found distinct and unique expression patterns of T-bet (Th1) and GATA-3 (Th2) mRNA in the genital tract in response to a *C. trachomatis* infection with a dominance of T-bet in the UGT and PALN, while GATA-3 was exclusively upregulated in the LGT, supporting that the UGT and LGT were functionally separate compartments.

### No or poor Th17 activity in the genital tract during *C. trachomatis* infection

Th17 is a recently identified T helper subset that has been ascribed important roles in tolerance, autoimmune diseases and infections [11], [20], [26]. This population of T helper cells remains poorly studied in the genital tract, and to date, its role in *Chlamydia* genital tract infection has not been described. The Th17 subset differentiates under the control of the transcription factor RORγ-t [15]. Thus, we used RT-PCR to report the presence of Th17 cells through the expression of RORγ-t mRNA during *Chlamydia* genital tract infection. The expression of RORγ-t mRNA in the draining lymph nodes did not significantly change from naïve levels during infection, although there was an initial decrease in expression from naïve levels observed in the ILN (Fig. 4C, D). During homeostatic conditions in the UGT of naïve mice, RORγ-t mRNA expression was low, whereas these levels were somewhat higher in the LGT (Fig. 4C, E). However, following infection, RORγ-t mRNA expression decreased in the LGT and UGT (Fig. 4E). These data suggested that the Th17 subset were not expanded in the genital tract in response to a *C. trachomatis* infection.

### Treg development in the genital tract during *Chlamydia* infection

**Figure 5:**
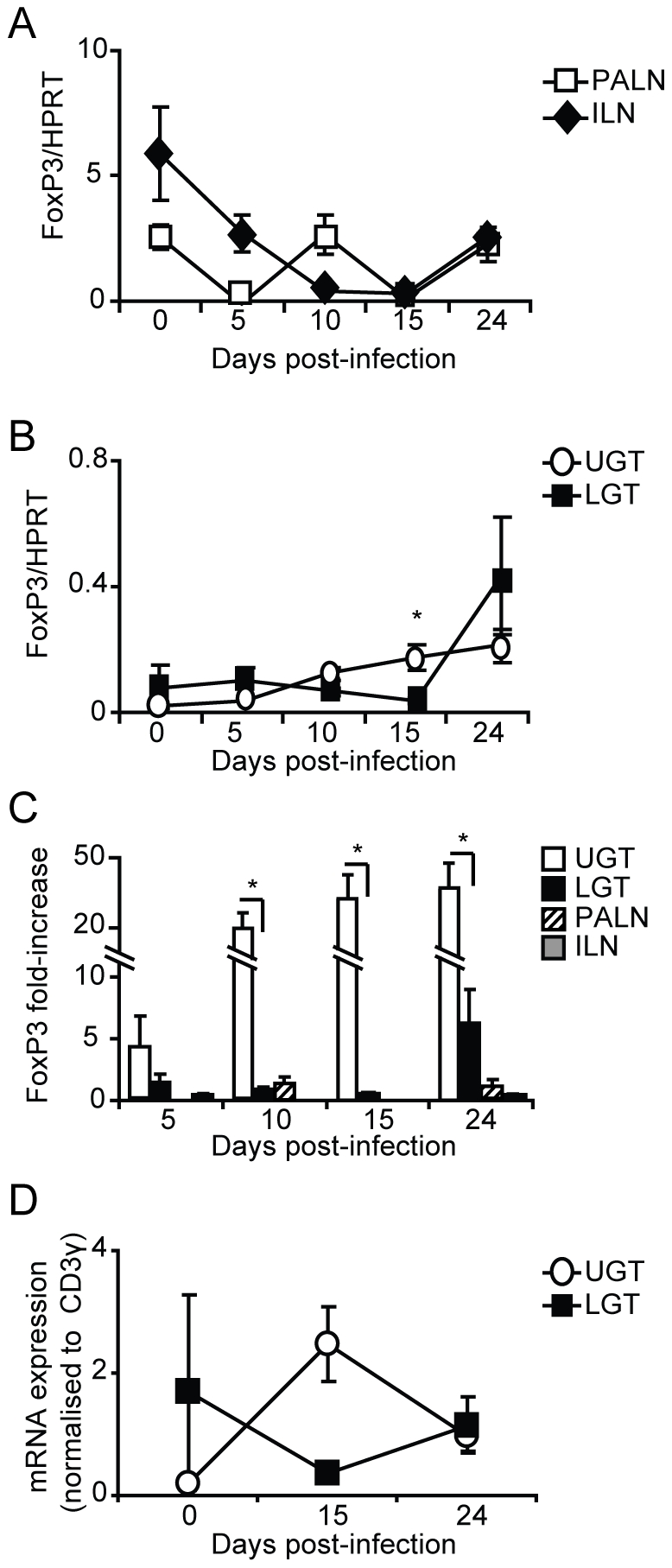
Infection induces Tregs in the genital tract. Using RT-PCR we determined the FoxP3 mRNA expression levels in PALN, ILN, upper (UGT) and lower genital tract (LGT) after infection with *C. trachomatis*. Expression of FoxP3 mRNA was normalized to the expression of the housekeeping gene, HPRT (A-B), or CD3-γ (D). Data is presented as mean expression ± SEM or (C) fold-increase over naïve levels. Values are from one representative experiment of 3 with similar results, with each experiment containing 5 mice per time point. * p<0.05.

The balance between CD4^+^ effector cell populations and Tregs is considered critical for limiting the immunopathological outcome of a chlamydial infection. We have recently shown that lack of FoxP3^+^ Tregs, as seen in the ICOS-deficient mice, increases the risk of developing severe immunopathology following a genital tract infection with *C. trachomatis* [23]. Here we found an increase in expression of FoxP3 mRNA in the LGT and UGT (Fig. 5B). FoxP3 mRNA levels in the UGT increased gradually from day 10, and by day 24 were at 30-fold of those observed in naïve mice, whereas levels in the LGT had increased 6-fold by day 24 (Fig. 5B, C). By contrast, RT-PCR analysis of the draining lymph nodes revealed little activity and only decreased FoxP3 mRNA levels followed upon infection (Fig. 5A). To conclude, we observed a relative increase in FoxP3 mRNA expression in the UGT, paralleling the increase in mRNA expression of T-bet (Fig. 5B). By contrast, the LGT, dominated by GATA-3 mRNA expression, and exhibited less of a change in FoxP3 mRNA in response to a genital tract infection with *C. trachomatis*.

### T cells from the ILN and PALN exhibit similar tissue homing properties

**Figure 6:**
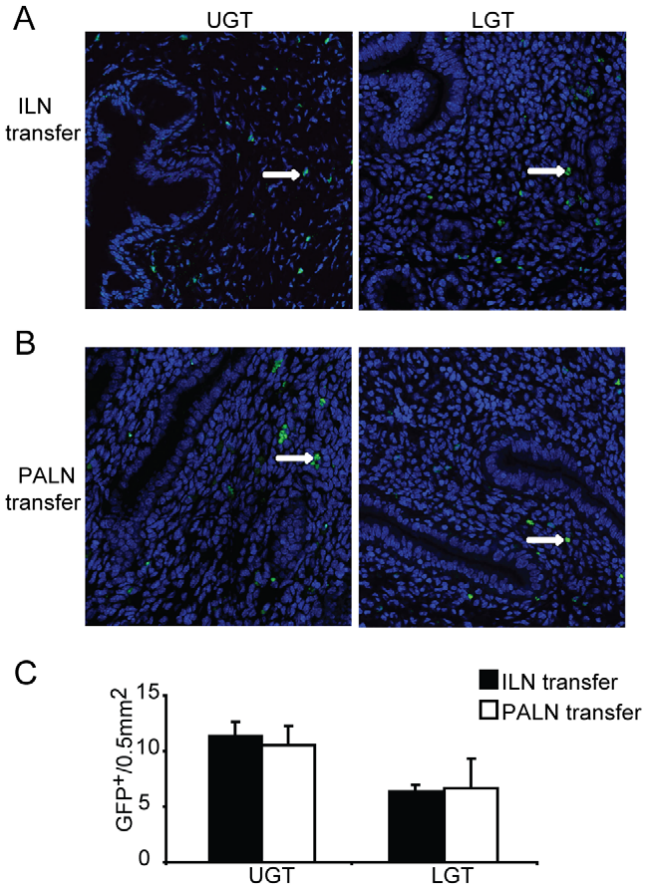
Local factors influence the accumulation of T helper subsets in the genital tract. GFP-expressing T cells were isolated from the (A) ILN or (B) PALN of mice infected with *C. trachomatis* 7 days earlier. GFP^+^CD4^+^ T cells were then adoptively transferred to recipient infected WT mice after 10 days of a primary infection. Upper genital tract (UGT; left panels) and lower genital tract (LGT; right panels) tissues were harvested 4 days after transfer. To-Pro-3 counterstained (blue) and GFP^+^ cells (white arrows;green) were visualized by confocal microscopy. (C) GFP^+^ cells were counted in 14 visual fields from at least 3 mice per group. Data represent the mean + SEM per 0.5 mm^2^ of tissue.

Given that we observed an association between enhanced T-bet mRNA expression in the UGT and the PALN, while ILN only weakly expressed T-bet and more distinctly GATA-3 mRNA, we hypothesized that imprinting of UGT CD4^+^ T cells could have occurred in the PALN rather than in the ILN. To investigate to what extent the differences in transcription factor mRNA expression in UGT and LGT reflected differential homing properties acquired by primed T cells in the PALN and ILN, we adoptively transferred GFP-transgene expressing T cells from either the ILN or PALN of infected or naïve mice into naïve recipient C57Bl/6 mice or mice that had been infected 10 days earlier. Irrespective of whether the CD4^+^ T cells were isolated from the PALN or ILN, we found T cells were able to home to both the UGT and the LGT of *Chlamydia*-infected mice, albeit more cells ended up in the UGT (Fig. 6A, B). Of note, GFP^+^ T cells could not be found in the genital tract of adoptively transferred naïve mice, indicating that homing of specific T cells to the genital tract does not occur in the absence of infection (data not shown). Therefore, CD4^+^ T cells acquire homing properties in the PALN or ILN that do not discriminate between UGT and LGT, suggesting that these two functionally distinct compartments appeared to be influenced by local factors in the respective tissues, rather than unique imprinting by antigen-presenting DC restricted to the PALN or the ILN. Thus, the anatomically distinct T helper subset profiles observed could have been influenced by local factors in the tissues.

### IL-10 production critically influences the lower genital tract

**Figure 7:**
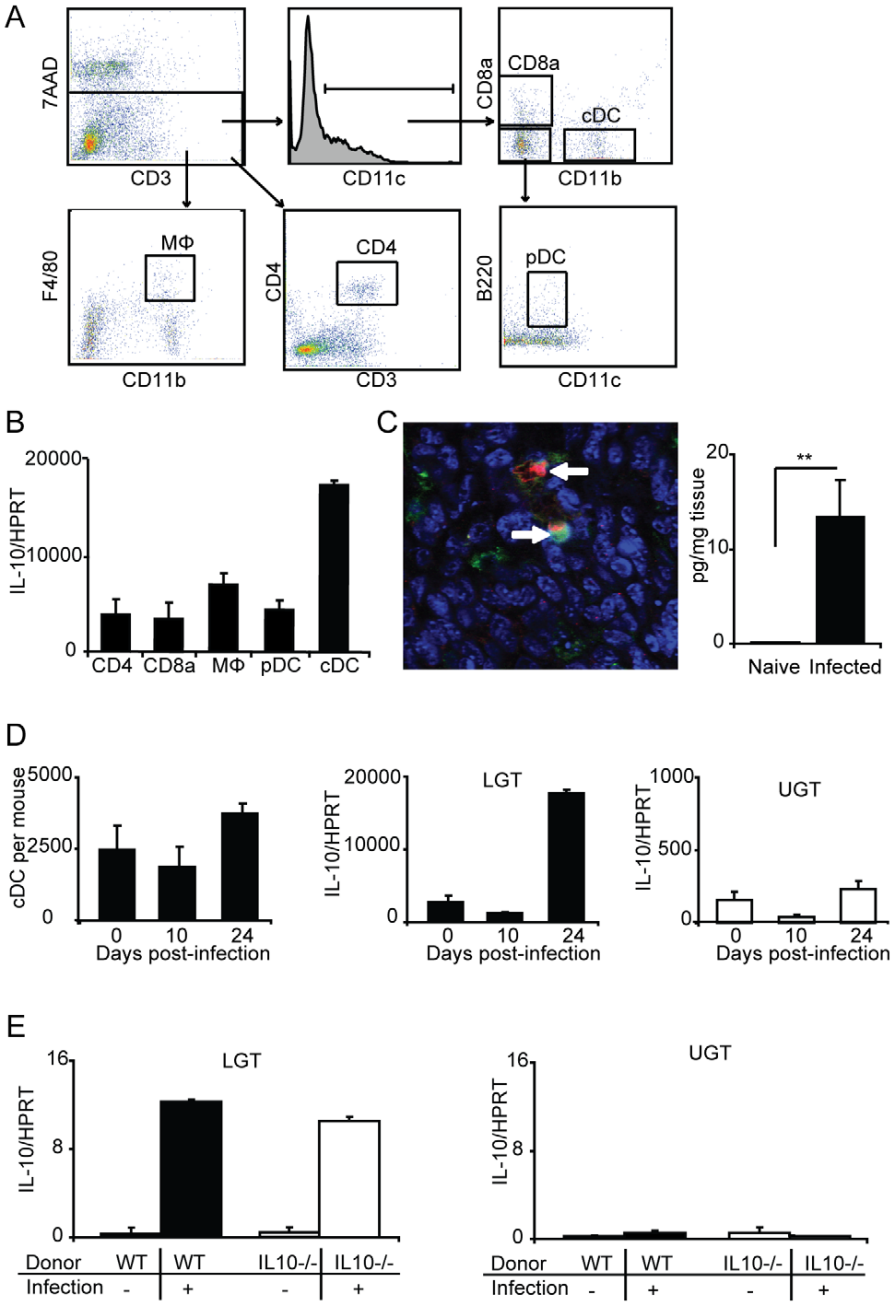
Lower genital tract DC are the primary source of IL-10 production following *C. trachomatis* infection. (A) Lymphocytes from the lower genital tracts (LGT) from naïve WT mice or *C. trachomatis* infected (day 24) were isolated and sorted into CD4, CD8α, MΦ, pDC, and cDC populations (see Materials and Methods for definitions). (B) The relative level of IL-10 mRNA expression in the indicated sorted populations was analyzed by RT-PCR on day 24 of infection. Values were normalized against the housekeeping gene (HPRT) and given as mean normalized expression ± SEM, from a representative of 2 experiments, each with 3 samples per group and 3 pooled mice in each sample. (C, left panel) Immunohistochemistry was used to confirm the presence of IL-10 protein (TxRed; red), CD11c (FITC;green) and Topro-3 (blue) on day 24 of infection. (C, right panel) IL-10 levels from the LGT of naïve mice or after 24 days of infection were measured using an ELISA. (D) The numbers of sorted cDC per mouse from the LGT of naïve mice, and on day 10 and 24 of infection (left panel). IL10 mRNA expression normalized to HPRT in the sorted cDC from the LGT (middle panel) and UGT (right panel) of naïve mice, and on day 10 and 24 of infection. (E) IL10^−/−^ or WT CD4^+^ T cells were adoptively transferred to nu/nu mice and IL10 mRNA expression in the LGT (left panel) or upper genital tract (UGT; right panel) was assessed in unchallenged mice or after 24 days of infection.

Since we observed a significantly increased level of IL-10 mRNA expression in the LGT on day 24 of the infection and that this cytokine is a particularly strong inhibitor of Th1-development we analyzed the possible source for the production of IL-10 in the tissue. To locate the cellular source of IL-10, we isolated distinct populations of cells, with purity of over 97%, from the genital tract of naïve mice and *Chlamydia* infected mice. Surprisingly, the major source of IL-10 mRNA in the LGT of infected mice was found to be the classical DC population (cDC; CD11b^+^CD11c^+^) (Fig. 7A, B). Labeling of frozen sections of LGT with anti-IL-10 also confirmed the presence of CD11c^+^ cells producing this cytokine on day 24 following infection (Fig. 7C). Moreover, after saponin extraction of cytokines in biopsies of LGT we could detect IL-10 by ELISA, but only in samples from infected mice (Fig. 7C). The high IL-10 mRNA expression in the cDC of the LGT was observed on day 24 after infection, while at earlier time points the IL-10 mRNA expression in LGT did not differ from that in naïve mice, although this was substantially higher levels than in UGT cDC. (Fig. 7D). Interestingly, on day 24 post-infection we also observed an increase in the size of the cDC population in the LGT (Fig. 7D). By contrast, only weak expression of IL-10 mRNA was found in macrophages (CD11b^+^F4/80^+^), plasmacytoid DC (pDC; CD11c^+^CD11b^-^CD19^-^B220^+^), CD8α^+^ DC (CD11c^+^CD8α^+^), CD4^+^ T cells (CD4^+^ CD3^+^), or epithelial sheets (Fig. 7B and data not shown).

To rule out that Tregs were directly or indirectly responsible for the IL-10 dominance in the LGT we undertook adoptive transfer experiments with CD4^+^ T cells from IL-10-deficient (IL-10^−/−^ ) or wild-type (WT) mice injected into nu/nu mice. Following a genital tract primary infection, nu/nu mice responded with IL-10 mRNA expression levels in the LGT of comparable magnitude irrespective of if the CD4^+^ T cells were IL-10-deficient or normal (Fig. 7E). As before, the IL-10 mRNA was restricted to the LGT and only expressed at low levels in the UGT of infected mice (Fig. 7E). This result clearly demonstrated that CD4^+^ T cells were not directly or indirectly responsible for the IL-10 production in the LGT. Taken together, the LGT mucosa appeared to be a privileged tissue through anti-inflammatory activity, provided by regulatory cDC producing IL-10. Hence, our data suggested that Th1-effector T cell activity in the LGT in response to a genital tract chlamydial infection was restricted by DCs.

### Failure in IL-10-deficient mice to maintain a privileged anti-inflammatory compartment in the lower genital tract

**Figure 8:**
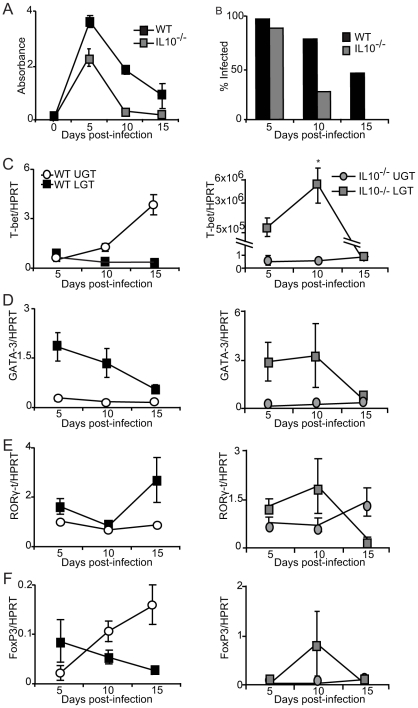
IL-10 production inhibits T-bet mRNA expression during infection. (A-B) IL-10^−/−^ and WT mice were infected intravaginally with *C. trachomatis* and bacterial shedding was followed using a MikroTrak EIA. (A) Results represent mean absorbance ± SEM and (B) frequency of infected mice per group per time-point. (C-F) Transcription factor mRNA expression was determined by PCR in the upper genital tract (UGT) and lower genital tract (LGT) from IL-10^−/−^ (right panels) and WT mice (left panels) at indicated time points after infection. Values were normalized against the housekeeping gene (HPRT) expression level and given as mean expression ± SEM. Values are from one representative experiment of 2 giving similar results, and 3 mice in each group per time point.

Finally, to test the notion that IL-10 was a locally produced factor responsible for establishing a privileged compartment in the LGT we undertook experiments in IL-10^−/−^ mice. Given that it has been reported that IL10^−/−^ mice display enhanced immunity to chlamydial infection, we asked if the LGT compartment was dominated by Th1 effector cells rather than Th2 cells, as seen in WT mice [25], [27]. To this end, IL-10^−/−^ mice were infected with *C. trachomatis* and the mRNA expression of transcription factors T-bet, GATA-3, RORγ-t and FoxP3 was analyzed by RT-PCR. We found that IL-10^−/−^ mice displayed enhanced clearance of infection, as reported earlier [27]. In fact, elimination of bacteria was complete by day 15 in IL-10^−/−^ mice, at a time point when more than 40% of WT mice remained infected (Fig. 8A, B). With regard to transcription factor expression we found that T-bet mRNA expression in the LGT of IL-10^−/−^ mice was strikingly up-regulated, representing a 50×10^6^-fold increase over WT levels at the same time point (Fig. 8C). This dramatic shift in T-bet and Th1-development was clearly a local consequence of lack of regulatory IL-10 in the LGT as the level of T-bet mRNA expression in the UGT was relatively unchanged in these mice (Fig. 8C). Also, GATA-3 and RORγ-t mRNA expression levels both in the UGT and LGT were relatively unaffected in the absence of IL-10, further supporting the notion that IL-10 in LGT renders this tissue a status of an anti-inflammatory privileged site (Fig. 8D, E). Interestingly, whereas we had observed that T-bet mRNA expression levels in the UGT of WT mice were accompanied by a corresponding increase in FoxP3 mRNA expression, this did not apply to the LGT in IL-10^−/−^ mice, where FoxP3 mRNA expression levels were not significantly changed (Fig. 8F). The absence of IL-10 did not change TGF-β mRNA expression levels in either the UGT or LGT compared to that seen in WT mice (not shown). These results favor that local production of IL-10 in response to a genital tract infection with *C. trachomatis* prevents the development of strong Th1-immunity in the LGT. Hence, we propose that cDC derived IL-10 provides conditions for a privileged compartment in the LGT.

## Discussion

In the present study we set out to learn more about the different effector and regulatory CD4^+^ T cell populations induced by a primary genital tract infection with *C. trachomatis*, an obligate intracellular bacterium. Unexpectedly we found that a key player in establishing the peripheral CD4^+^ T cell repertoire in the local genital tract tissue was a population of classical CD11c^+^ CD11b^+^ DCs, which produced significant levels of IL-10. This production appeared to prevent Th1-dominance in the LGT, whereas the latter was clearly the main CD4^+^ T cell population in the UGT. Hence, we propose that anti-inflammation prevails in the LGT as a consequence of regulatory cDCs producing IL-10. This could possibly impair anti-infectious effector functions, while preventing unwanted tissue destruction exerted by the Th1 cells during a *Chlamydia*-infection. The LGT could, thus, be viewed as a privileged site.

Here we report a functional distinction between the LGT and UGT, driven by a local cytokine production, as immune protection against a genital tract infection develops. Surprisingly, we found that IL-10 played a critical role for this effect. The evidence for that was derived from experiments in IL-10^−/−^ mice where this functional dichotomy was completely lost and both tissues were dominated by CD4^+^ Th1-cells. Furthermore, it was clear that imprinting of homing abilities in newly primed CD4^+^ T cells in the draining lymph nodes, ILN or PALN, did not support such a dichotomy, since irrespective of their origin, CD4^+^ T cells were found in both UGT and LGT. Rather, it appeared that the IL-10 production in the LGT influenced the accumulation and/or differentiation of Th2 cells and prevented the expansion of Th1 cells in that tissue. Furthermore, adoptive transfer experiments showed that the ability to produce IL-10 was not a critical property of the CD4^+^ T cells as such since IL-10-deficient cells in nu/nu mice did not alter the functional dichotomy between LGT and UGT seen in WT mice. Collectively our data suggest that genital tract cDC could exert a regulatory function by influencing the local CD4^+^ T cell repertoire in the LGT in response to a genital tract chlamydial infection.

CD4^+^ T cells are of fundamental importance for protection against *C. trachomatis* genital tract infections, yet a detailed understanding of functional qualities of CD4^+^ T cell subsets during infection is limited [5], [6], [18]. Such studies have been technically demanding especially because of the difficulties in isolation of T cells from the tissues [28]. To circumvent this problem, we developed RT-PCR assays to detect mRNA expression of transcription factors and cytokines in the genital tract. Hence, for the first time, using very specific and sensitive methods, we were able to follow global CD4^+^ T cell differentiation and immune regulation in the genital tract in response to a *C. trachomatis* infection. Quite unexpectedly, we found evidence that unraveled a complex system of expansion and regulation of CD4^+^ T helper subsets in the UGT and LGT, establishing a clear functional dichotomy between the two compartments. Whereas, a strong Th1 profile was induced in the UGT with an exclusive presence of IFN-γ, the LGT appeared to be a privileged site with anti-inflammatory IL-10 and a dominance of Th2 cells. Our initial theory was that the dichotomy between LGT and UGT was established by imprinting different homing properties on CD4^+^ T cells by antigen-presenting cDC in the regional lymph nodes. This was because we observed an up-regulation of T-bet mRNA in the PALN and of GATA-3 mRNA in the ILN we thought that a differential homing pattern could have explained the selective enrichment of Th1 and Th2 cells in the UGT and LGT, respectively. However, this notion was not supported by our finding that T cells from both PALN and ILN were capable of homing to the UGT and LGT.

Rather, an alternative explanation for the dichotomy was considered, namely that local production of regulatory cytokines in the LGT promoted Th2 cells and largely prevented Th1 cells in this tissue. This latter theory was also supported by two observations; i) that local cDC in the LGT produced IL-10 and that ii) in IL-10 deficient mice the LGT environment was changed and instead hosted a dominant Th1 cell population in response to the genital tract infection. Moreover, primed and tissue migrating CD4^+^ T cells, including Tregs, were not required to produce IL-10 to allow for the dichotomy between LGT and UGT. The importance of local production of immune regulating factors in the genital tract is supported by findings reported by Maxion *et al*., who showed that chemokines associated with Th1 responses, namely CXCL10, CXCL9 and CCL5 were found exclusively in the oviducts, while the Th2-associated chemokine CCL11 was elevated primarily in the cervical region following infection [29]. However, to accommodate our results with these findings we must assume that the selective expression pattern of chemokines in the LGT is influenced by IL-10 and that in the absence of this cytokine the expression of CXCL10, CXCL9 and CCL5 prevails also in the LGT, allowing for the migration and accumulation of Th1 cells in both UGT and LGT, in agreement with what we observed in IL-10-deficient mice. Indeed, previous studies have documented that endogenous IL-10 plays a crucial down-modulating role on both CC and CXC chemokine expression and neutrophil influx, in e.g lung and gut intestinal tissues [30], [31]. Moreover, several reports associate CXCL10 expression with tissue-recruitment of Th1 cells and IL-10 production strongly inhibits CXCL10 expression [32], [33]. Hence, we propose that local IL-10 production by cDC is the key factor in maintaining LGT an anti-inflammatory privileged site, down-modulating CXCL10 and preventing Th1 cell influx. Whether withdrawal of IL-10 in the LGT, as in IL-10^−/−^ mice, allows for CXCL10, CXCL9 and CCL5 chemokine production to increase will be investigated in future studies.

In the present study we have documented IL-10 producing cDC in the LGT, which appeared to control the distribution of CD4^+^ effector T cells and secured that the LGT was a privileged compartment during *C. trachomatis* infection. However, to unequivocally document that IL-10 producing cDC in the LGT were responsible for the lack of Th1 cells and dominance of Th2 and GATA3-expressing cells in the LGT, we would have to engineer a mouse model where these cells could be selectively depleted. Unfortunately, there exists no such model at present given that depletion of cDC in general in the CD11c-DTR (diphtheria toxin receptor) mouse model would take away all DCs, leaving no DCs for priming of a T cell response, plus the fact that repeated injection of DT would be required, which also depletes other cell subsets, including plasma blasts, activated CD8^+^ T cells, NK cells and some populations of macrophages [34], [35], [36], [37]. We are, therefore, currently exploring the possibility to generate a mouse model with IL-10-deficiency in the cDC population through mating mice with a loxP IL-10 gene with mice CD11c-cre mice.

We found evidence that not only CD4^+^ effector T cells, but also Tregs, may be differentially distributed to the UGT and LGT. We found that increases in FoxP3 mRNA expression occurred in the UGT already 10 days after inoculation, suggesting that early Th1 migration into the UGT promoted the establishment of FoxP3^+^ Tregs in this tissue. Whereas there was a clear association between increases in Th1 cells and Tregs in the UGT, such a pattern was not as clear for the LGT, as the RT-PCR detection revealed only 6-fold increases in FoxP3 mRNA in the LGT as compared to nearly 40-fold increases in the UGT over the course of the infection. Functionally, this observation agrees well with many previous reports because Tregs could effectively dampen the Th1 activity and protect against tissue damage [38]. As IL-10 from cDC appears to be the main anti-inflammatory factor in the LGT, Tregs could fulfill this function in the UGT, again pointing to the dichotomy and very compartmentalized functions of the LGT and UGT. At present we can only speculate that the mechanism by which Tregs limit immunopathology in the UGT is through TGF-α or other mechanisms rather than IL-10, since IL-10 mRNA levels were low in the UGT [39].

Studies of Th17 cells have largely demonstrated their involvement in autoimmunity and recently also their role in host defense against bacteria [20], [21], [40], [41]. Th17 cells are induced in a number of bacterial infections including *Salmonella* e*nteritidis*, *Helicobactor pylori*, and *Mycobacterium tuberculosis* [21], [41], [42], [43]. Although there are 6 members of the IL-17 family, Th17 cells produce large amounts of IL-17A and, therefore, most effector functions of Th17 cells have been attributed to the production of this cytokine. Th17 cells are induced by the cytokines TGF-β and IL-6, which have both been shown to be produced in response to *C. trachomatis* [44], [45], [46]. Contrary to other studies of bacterial infections we failed to detect any major alterations in the Th17 subset in either the UGT or the LGT, as assessed by detection of mRNA for RORγ-t. We must, therefore, conclude that this subset appears not to play a role in host protection against a genital tract infection with *C. trachomatis*. This result is also at variance with a recent study by Bai *et al*., who reported on lung infections induced by *Chlamydia muridarium*, the mouse-specific *Chlamydia* species. These authors treated mice with neutralizing anti-IL17 antibodies and found poor immunity to infection [19]. As IL-17 has not been found to inhibit chlamydial growth the effect of anti-IL17 antibody treatment was rather attributed to poor IL-12 and strong IL-10 production by DC, leading to reduced Th1-effector functions [47]. Similar to the chlamydial lung infection, the Th17 response in *M. tuberculosis* infections is induced rapidly and is necessary to attract Th1 cells to the lung to enhance the adaptive immune response [43]. Although, we observed some increases in IL-17 mRNA in the LGT by day 24 following infection a concomitant increase in RORγ-t mRNA was not observed. Hence, we do not think Th17 cells are prominent in chlamydial genital tract infections. It should be emphasized that the Th17 subset is not the only cell type capable of producing IL-17; γδ^+^ T cells [48], NKT cells [49], neutrophils [50] and even FoxP3^+^ T cells [51] have also been shown to produce this cytokine. However, because of the complexity of the CD4^+^ T cell response that we observed a similar type of anti-IL17 antibody treatment experiment should be performed to rule out any involvement of IL-17 in protective immunity against *C. trachomatis* genital tract infections.

Recently, Moniz *et al.* described two subsets of DC in the mouse genital tract in response to *C. muridarium* infection [52]. These authors found that DC primed Th1 cells, while pDC produced IL-6 and IL-10 and primed non-Th1 cells. Our findings are somewhat T at variance with this observation, in that pDC from the LGT did not produce significant levels of IL-10, while cDC did. However, the cDCs of the LGT may represent a subset of cells resembling those found in the gut intestine. Recent elegant studies by Varol *et al.* have documented several subsets of lamina propria DCs, especially a population of non-monocyte-derived CX_3_CR1^-^ CD103^+^ were reported critical for homeostasis, whereas a monocyte derived DC population failed to control inflammation [53]. Also in the Peyer's patches, a DC subset has been reported which preferentially secretes IL-10 and generates Th2 responses [54]. By contrast, splenic DC produce IL-12 and favor Th1 generation. In agreement with this latter notion, Th1 instead of Th2-responses were induced in IL-10^−/−^ mice. Th1 polarization of the immune response in the absence of IL-10 correlated also with better protection. IL-10 producing cDCs following chlamydial infection of the lung have been shown to reduce allergen-specific cytokine production and CD4^+^ T cell responses [55]. In the lung infection model of *C. muridarium,* adoptively transferred DCs prevented Th1 cell expansion, indicating that DC in chlamydial infections have a regulatory function [19]. These DC produced high levels of IL-10, which resulted in poor Th1 expansion and poor clearance of bacteria from the lung [19].

The most important observation in the present study was that IL-10 production by cDC coincided with a lack of Th1 expansion in the LGT. Previous studies have reported that in IL-10^−/−^ mice, resistance to *Chlamydia* was associated with early maturation and activation of DCs in the draining lymph nodes, enhanced antigen presentation and stimulation of increased IFN-γ production from the T cells [25]. Our present data complement these findings by showing that IL-10 strongly regulates the presence of Th1 cells in the LGT. Interestingly, this effect was restricted to Th1 functions, since no changes in Th2 or Th17 transcription factor mRNA expression were observed in the LGT of IL-10^−/−^ mice. Whether these regulatory cDC migrated into the tissue or were resident in the LGT as the genital tract *C. trachomatis* infection ascended is not known. As aforementioned, the literature support for that they were derived from monocytes is weak as such DCs have nearly always been associated with pro-inflammatory responses [53]. Rather, it appears that the LGT cDC are bone-marrow derived and at least three subsets of these cells have been described in the mouse vaginal epithelium [56]. We believe that a better understanding of the functional dichotomy between UGT and LGT and the role of cDC-derived IL-10 in regulating the CD4^+^ T cell repertoire in the LGT is of vital importance to the development of future effective and safe local vaccines against *C. trachomatis*. Such knowledge could also have important implications for how to prevent the immunopathology associated with genital tract chlamydial infections.

## Materials and Methods

### *Chlamydia* stocks

A human genital tract clinical isolate of *C. trachomatis* serovar D was propagated in buffalo-green monkey kidney cells and purified by centrifugation. Chlamydia IFU were enumerated using the method described below and stored in sucrose–phosphate–glutamate (SPG) buffer at −80°C. The infectivity of *C. trachomatis* stocks of elementary bodies (EBs) was tested by intravaginal infection of C57BL/6 mice using a range of doses. We found that 10^6^ inclusion forming units (IFU) was required for 100% of the mice to be infected on day 8.

### Mice

6–8 week old female C57BL/6 mice were purchased from Taconic (Denmark). IL10^−/−^ mice were bred at the Department of Experimental Biomedicine at the University of Gothenburg, Sweden [57]. UBI-GFP/BL6 mice [58], which express a transgene coding for green fluorescent protein (GFP) under control of the human ubiquitin C promoter, were used for transfer experiments. Nu/nu mice were purchased from Taconic (USA). All experiments include DepoProvera treated (2.5 mg subcutaneously 7 days prior to analysis) naïve controls of each group.

### Ethics statement

Mice were maintained under specific pathogen-free conditions, according to FELASA specified guidelines, at the Department of Experimental Biomedicine at the University of Gothenburg, Sweden. Approval was obtained from Swedish Animal Welfare Agency.

### Bacterial infection and challenge protocols

Mice were given 2.5 mg subcutaneous injection of medroxyprogesterone acetate (DepoProvera, Pharmacia Sverige AB) 7 days prior to the inoculation of approximately 1×10^6^ inclusion forming units (IFU), 10^5^ IFU, or 10^6^ heat-killed IFU of *C.* trachomatis elementary bodies (EBs) intravaginally. Four weeks after the resolution of the primary infection, the inoculation procedure was repeated in a manner identical to that described for the primary infection. Bacterial shedding was monitored at 2, 4, 8, 16 and 24 days post-reinfection.

### Detection of chlamydial infection

Detection of *C. trachomatis* infection was performed in two ways. Firstly, bacterial shedding was monitored at 8-day intervals using a commercial MicroTrak II *Chlamydia* EIA kit (Trinity Biotech plc.) according to manufacturer's instructions. Samples with an absorbance greater than the provided cut-off value were considered positive for chlamydial shedding. This detection method correlates closely with assessment of inclusion forming units (IFU) as described [2].

To confirm the level of infection at each time point, the number of IFU were enumerated by infection of HeLa cell monolayers, as previously described [59]. Swabs were collected in SPG buffer, vortexed and centrifuged at 13000 rpm for 10 minutes. Samples were then sonicated for 30 seconds. EBs were then added to HeLa cell monolayers and centrifuged at 280 ×*g* at room temperature for 1 hour followed by incubation at 37°C for 30 minutes. Plates were washed with 1× with HBSS and culture medium containing cycloheximide (2 µg/ml) was added. After 40 hours incubation at 37°C in a 5% CO_2_, infected monolayers were fixed by addition of 100 µl/well of methanol at room temperature for 20 minutes. The infected monolayers were stained with biotin-conjugated anti-MOMP antibody (Abcam), followed by streptavidin-alkaline phophatase (Dako), and developed by the addition of 1-Step NBT/BCIP reagent (Pierce). The reaction was stopped by rinsing with water, and the plates were allowed to air dry before counting.

### PCR analysis

The uterus/uterine horns (upper genital tract; UGT), the vagina/cervix (lower genital tract; LGT), para-aortic lymph nodes (PALN) and inguinal lymph nodes (ILN) were collected at the indicated time points. Tissues were stored in RNAlater (Qiagen) before total mRNA was isolated using Qiagen homogenizer and RNAeasy minicolumns (Qiacube, Qiagen) according to manufacturer's instructions. The resulting extraction was used for cDNA synthesis using oligo (dT) primer and SuperScript RT (Invitrogen Life Technologies) and analyzed by RT-PCR. Primers (MWG-biotech) used for the determination of transcription factor mRNA levels using SYBR green technology were as follows: GATA-3 forward (5′- CTT ATC AAG CCC AAG CGA AG -3′), GATA-3 reverse (5′- CCC ATT AGC GTT CCT CCT C -3′), T-bet forward (5′- TCAACCAGCACCAGACAGAG -3′), T-bet reverse (5′- AACATCCTGTAATGGCTTGTG -3′), Foxp3 forward (5′- AGC TGG AGC TGG AAA AGG A -3′), Foxp3 reverse (5′- GCT ACG ATG CAG CAA GAG C -3′), IFN-γ forward (5′- GCC ATC AGC AAC AAC ATA AGC -3′), IFN-γ reverse (5′- TGA GCT CAT TGA ATG CTT GG -3′), IL-10 forward (5′- GCT CCT AGA GCT GCG GAC T -3′), IL-10 reverse (5′- TGT TGT CCA GCT GGT CCT TT -3′), IL-4 forward (5′- CATCGGCATTTTGAACGAG -3′), IL-4 reverse (5′- CGAGCTCACTCTCTGTGGTG -3′), IL-17A forward (5′- TGT GAA GGT CAA CCT CAA AGT C -3′), IL-17A reverse (5′- AGG GAT ATC TAT CAG GGT CTT CAT T -3′) RORγ-t forward (5′- GGT GAC CAG CTA CCA GAG GA -3′), RORγ-t reverse (5′- CCA CAT ACT GAA TGG CCT CA -3′), TGF-β forward (5′- CAC CGG AGA GCC CTG GAT A -3′), TGF-β reverse (5′- TTC CAA CCC AGG TCC TTC CTA -3′), CD3□ forward (5′- CCAAGGAAACCAACTGAGGA -3′), CD3□reverse (5′- TTGATTCTGGGTGCTGGATAG -3′), HPRT forward (5′- TCC TCC TCA GAC CGC TTT T -3′), HPRT reverse (5′- CCT GGT TCA TCA TCG CTA ATC -3′) . RT-PCR was performed using the LightCycler System and Relative Quantification software (Roche Diagnostics, GmbH). Results were expressed as a normalized ratio of the target mRNA to housekeeping mRNA.

### Immunohistochemistry

The reproductive tract, including the uterus/uterine horns (upper genital tract; UGT) and the vagina/cervix (lower genital tract; LGT), was removed, snap frozen in TissueTek OCT Compound (Histolab Products AB) and stored at –80°C within 2 hours. Cryostat sections of 7 µm were fixed in acetone before blocking with 0.3% H_2_O_2_, blocking using an avidin-biotin blocking kit (Vector Laboratories), followed by 20% normal horse serum. Sections were incubated with anti-CD4-biotin (BD PharMingen) followed by anti-rat IgG and developed using peroxidase-conjugated avidin (DAKO Cytomation) and a commercial peroxidase AEC substrate (Sigma-Aldrich). Sections were counterstained with HTX and mounted with Faramount (Histolab Products AB). For intracellular cytokine staining, sections were fixed as above and permeabilised in 0.1% saponin/PBS. Sections were incubated with biotin-conjugated anti-IL-10 or FITC-conjugated anti-IFN-γ and biotin-conjugated anti-CD4 or FITC-conjugated anti-CD11c (BD Pharmingen), followed by streptavidin-conjugated TxRd (Vector) and Topro-3 (Invitrogen). Negative controls were stained with isotype-matched irrelevant antibodies or the secondary antibody in absence of a primary antibody. Sections were visualised using a Leica LSC microscope or Zeiss LSM 510 Meta confocal microscope.

### T cell transfers

The draining lymph nodes (ILN and PALN) of the genital tract were harvested from UBI-GFP/BL6 mice, after 7 days of infection with *C. trachomatis*. CD4^+^ T cells were purified by negative selection using MACS. Briefly, single cell suspensions were prepared and incubated with CD4^+^ T cell biotin antibody cocktail (Miltenyi Biotec) for 10 minutes at 4°C, followed by biotin beads for 15 minutes. The cell suspension was transferred to a CS MACS column according to the manufacturer's instructions (Miltenyi Biotec). Purity of eluted T cells was controlled using FACS (typically around 90%). 1×10^6^ cells were injected i.v. into recipient DepoProvera-treated C57BL/6 mice either before or after 10 days of infection. For nu/nu experiments, CD4^+^ T cells were isolated as describe from naïve IL10^−/−^ or C57BL/6 (IL-10^+/+^) mice and 1×10^6^ cells were injected i.v. into recipient DepoProvera-treated nu/nu mice. One day later, two groups of nu/nu mice were infected with *C. trachomatis*, while two remained uninfected controls.

### Isolation of genital tract lymphocytes

Isolation of lymphocytes from the UGT and the LGT was performed as previously described [60]. Briefly, the genital tract tissue was washed in calcium- and magnesium-free HBSS (CMF-HBSS; Life Technologies), supplemented with 25 mM HEPES (Life Technologies), and then incubated at 37°C in CMF-HBSS containing 5 mM EDTA (Merck) and 10% heat-inactivated horse serum (Life Technologies). Following each incubation, the supernatant containing the sloughed epithelial cells and the intraepithelial lymphocytes were collected, centrifuged and prepared for RNA isolation. For isolation of the mucosal lymphocytes the remaining tissue was incubated three times for 60 minutes with collagenase D (120IU/ml; Sigma-Aldrich) dissolved in RPMI 1640 containing 25 mM HEPES and 20% inactivated horse serum (Life Technologies). Cell suspensions were washed and stained with anti-CD11c, anti-CD11b, anti-MHCII, anti-CD4, and anti-CD8 (BD Pharmingen) for 30 minutes on ice. The cells were then washed twice with PBS containing 0.1% BSA and sorted using a FACSAria (BD biosciences). Sorted cells were sorted into PBS/0.1% BSA, immediately centrifuged and resuspended in 350 µl buffer RLT (Qiagen) for subsequent RNA extraction as described above. Sorted populations were defined as following: CD4; CD4^+^CD3^+^, CD80^+^; CD11c^+^CD8α^+^, MΦ; CD11b^+^F480^+^, pDC; CD11c^+^CD11b^-^CD19^-^B220^+^, cDC; CD11b^+^CD11c^+^.

### Measurement of cytokine production

Cytokine levels were measured from genital tract tissue or from *in vitro* stimulated sorted CD4^+^ T cells. Briefly, tissues were weighed and immediately placed in 10 volumes (wt/vol) of a protease inhibitor cocktail containing 10 mM EDTA, 2 mM PMSF, 0.1 mg/ml soybean trypsin inhibitor, 1.0 mg/ml BSA, PBS, pH 7.0. Tissues were incubated at 2% saponin at 4°C over night. Samples were clarified by centrifugation at 13000× *g* for 10 minutes at 4°C. Protein concentration was determined using a cytometric bead array (CBA; BD biosciences) or ELISA (BD biosciences). For sorted cells, CD4^+^ T cells were incubated at 37°C, 5% CO_2_, for 72 hours in the presence of PMA (Sigma) at 10 ng/mL and ionomycin (Sigma) at 1 µg/mL. Cell culture supernatants were analyzed by cytometric bead array (CBA) according to the manufacturer's instructions (BD biosciences) or by intracellular cytokine staining for analysis by flow cytometry.

### Intracellular cytokine staining

CD4^+^ T cells isolated from the genital tract of infected or naïve mice were stimulated with PMA/ionomycin for 3 days as described above. Cell suspensions were incubated for the final 5 hrs at 37° in the presence of 5 µg/ml Brefeldin A (Sigma-Aldrich). Cells were stained for surface molecules, fixed with 2% formaldehyde (HistoLab Products AB) and re-suspended in permeabilization buffer containing HBSS, 0.5% bovine serum albumin (BSA), 0.5% saponin and 0.05% azide. FITC-conjugated anti-IL-4 or IL-5 were added. Cells were detected using an LSRII flow cytometer (BD Biosciences) with diva software (BD Biosciences). Data were analysed using Flowjo software (Tree Star Inc).

### Statistical analysis

Mann-Whitney or Dunnett's C non-parametric tests were used for analysis of significance. *p<0.05, **p<0.01 denotes statistically significant differences.

### Gene IDs for proteins mentioned in the text

The following are the GeneIDs (Database: Entrez Gene) for each gene analyzed in this manuscript, given as gene name (official symbol GeneID: #): T-bet (Tbx21 GeneID: 57765); GATA-3 (Gata3 GeneID: 14462); RORγ-t (Rorc GeneID: 19885); FoxP3 (Foxp3 GeneID: 20371); IFN-γ (Ifng GeneID: 15978); IL-17A (Il17a GeneID: 16171); IL-10 (Il10 GeneID: 16153); IL-4 (Il4 GeneID: 16189); CD3γ ( Cd3g GeneID: 12502), HPRT ( Hprt1 GeneID: 15452).
